# Moderate hypothermia inhibits microglial activation after traumatic brain injury by modulating autophagy/apoptosis and the MyD88-dependent TLR4 signaling pathway

**DOI:** 10.1186/s12974-018-1315-1

**Published:** 2018-09-20

**Authors:** Fengchen Zhang, Haiping Dong, Tao Lv, Ke Jin, Yichao Jin, Xiaohua Zhang, Jiyao Jiang

**Affiliations:** 1 Department of Neurosurgery, Ren-Ji Hospital, School of Medicine, Shanghai Jiao Tong University, No. 160 Pujian Road, Shanghai, 200127 People’s Republic of China; 2 Department of Anesthesiology, Ren-Ji Hospital, School of Medicine, Shanghai Jiao Tong University, No. 160 Pujian Road, Shanghai, 200127 People’s Republic of China

**Keywords:** Apoptosis, Autophagy, Microglial activation, Toll-like receptor, Traumatic brain injury

## Abstract

**Background:**

Complex mechanisms participate in microglial activation after a traumatic brain injury (TBI). TBI can induce autophagy and apoptosis in neurons and glial cells, and moderate hypothermia plays a protective role in the acute phase of TBI. In the present study, we evaluated the effect of TBI and moderate hypothermia on microglial activation and investigated the possible roles of autophagy/apoptosis and toll-like receptor 4 (TLR4).

**Methods:**

The TBI model was induced with a fluid percussion TBI device. Moderate hypothermia was achieved under general anesthesia by partial immersion in a water bath for 4 h. All rats were killed 24 h after the TBI.

**Results:**

Our results showed downregulation of the microglial activation and autophagy, but upregulation of microglial apoptosis, upon post-TBI hypothermia treatment. The expression of TLR4 and downstream myeloid differentiation primary response 88 (MyD88) was attenuated. Moderate hypothermia reduced neural cell death post-TBI.

**Conclusions:**

Moderate hypothermia can reduce the number of activated microglia by inhibiting autophagy and promoting apoptosis, probably through a negative modulation between autophagy and apoptosis. Moderate hypothermia may attenuate the pro-inflammatory function of microglia by inhibiting the MyD88-dependent TLR4 signaling pathway.

**Electronic supplementary material:**

The online version of this article (10.1186/s12974-018-1315-1) contains supplementary material, which is available to authorized users.

## Background

In developing countries, traumatic brain injury (TBI) is a major cause of morbidity and mortality. The pathological process of TBI is quite complicated and is commonly divided into two phases, primary and secondary injury. The activation of resident microglia plays a key pro-inflammatory role in the acute secondary phase post-TBI [1–3].

Autophagy is a highly conserved intracellular process, which includes the degradation of abnormal accumulations of toxic substances, proteins, and damaged organelles, so that the proteins and other substances can be recycled efficiently. After TBI, autophagy can both protect cells and damage them [4–6]. In our previous study, we found that TBI could induce autophagy and apoptosis in neurons and glial cells. However, in the acute phase of TBI, hypothermia plays a cytoprotective role. Furthermore, we demonstrated that post-TBI hypothermia could upregulate the autophagy pathway, modulate apoptosis, and reduce cell death in neurons and glial cells; a possible mechanism for this is the negative regulatory effect of autophagy on apoptosis [7, 8]. We also found autophagy induction in microglia after TBI [9], but the regulatory effect of moderate hypothermia on microglial activation remains unknown.

Among the variety of receptors involved in microglial activation, toll-like receptors are significant for microglial activation and functioning. Toll-like receptor 4 (TLR4) is mainly expressed in microglia. TLR4 activates interleukin-1 receptor-associated kinase (IRAK) through the myeloid differentiation primary response 88 (MyD88)-dependent pathway; this further activates tumor necrosis factor receptor-associated factor 6 (TRAF6), which is followed by the activation of the downstream transcription factors nuclear factor kappa light chain enhancer of activated B cells (NF-κB), activator protein 1, and interferon regulatory factor-5. These transcription factors induce the expression of pro-inflammatory cytokines, such as interleukin-6 (IL-6), tumor necrosis factor alpha (TNF-α), and interleukin-12 (IL-12) [10–13].

In this study, we evaluated the effect of hypothermia on the microglial activation post-TBI and preliminarily explored the possible mechanisms, with regard to the relationship between autophagy, apoptosis, and the MyD88-dependent TLR4 pathway.

## Methods

### Animals and experimental design

All animal procedures in this study were approved by the Animal Care and Experimental Committee of the School of Medicine of Shanghai Jiao Tong University. Adult male Sprague–Dawley rats (280–300 g) were used. Rats were randomly divided into four groups: sham injury with normothermia group (SNG; 37 °C; *n* = 60), sham injury with hypothermia (SHG; 32 °C; *n* = 60), TBI with normothermia group (TNG; 37 °C; *n* = 60), and TBI with hypothermia group (THG; 32 °C; *n* = 60). Rats were housed in individual cages in a temperature- and humidity-controlled animal facility with a 12-h light/dark cycle. Rats were housed in the animal facility for at least 7 days before surgery, and they were given free access to food and water during this period.

### Surgical preparation

Rats were anesthetized through intraperitoneal injection (i.p.) of 10% chloral hydrate (3.3 mL/kg) and were then mounted in a stereotaxic frame. An incision was made along the midline of the scalp, and a 4.8-mm diameter craniectomy was performed on the left parietal bone (midway between the bregma and the lambda). A rigid plastic injury tube (a modified Leur-loc needle hub, with an inside diameter of 2.6 mm) was secured over the exposed, intact dura by using cyanoacrylate adhesive. Two skull screws (2.1 mm in diameter, 6.0 mm in length) were placed in the burr holes, 1 mm rostral to the bregma and 1 mm caudal to the lambda. The injury tube was secured to the skull with dental cement. Bone wax was used to cover the open needle hub connector after the dental cement had hardened (5 min). The scalp was closed with sutures. The animals were returned to their cages for recovery.

### Lateral fluid percussion brain injury

A fluid percussion device (VCU Biomedical Engineering, Richmond, VA) was used to cause TBI, as described in detail previously [10, 14]. The rats were subjected to TBI 24 h after the surgical procedure to minimize possible confounding factors of the surgery. In brief, the device consisted of a Plexiglas cylindrical reservoir filled with 37 °C isotonic saline. One end of the reservoir had a rubber-covered Plexiglas piston mounted on O-rings, and the opposite end had a pressure transducer housing with a 2.6-mm inside diameter male needle hub opening. On the day of the TBI, rats were anesthetized with 10% chloral hydrate (3.3 mL/kg, i.p.) and endotracheally intubated for mechanical ventilation. The suture was opened, and the bone wax was removed. The rats were disconnected from the ventilator, and the injury tube was connected to the fluid percussion cylinder. A fluid pressure pulse was then applied for 10 ms directly onto the exposed dura to produce a moderate TBI (2.1–2.2 atm). The injury was delivered within 10 s after disconnection from the ventilator. The resulting pressure pulse was measured in atmosphere by using an extracranial transducer (Statham PA 85-100; Gloud, Oxnard, CA) and recorded on a storage oscilloscope (Tektronix 5111; Tektronix, Beaverton, OR). After the initial observation, the rats were ventilated with a 2:1 nitrous oxide/oxygen mixture and the rectal and temporal muscle temperatures were recorded. The needle hub, screws, and dental cement were then removed from the skull, and the scalp was sutured closed. The rats were extubated as soon as spontaneous breathing was observed. The SNG and SHG rats were subjected to the same anesthetic and surgical procedures as the rats in the other groups but without being subjected to injury.

### Manipulation of temperature

The frontal cortex brain temperature was monitored with a digital electronic thermometer (model DP 80; Omega Engineering, Stamford, CT) and a 0.15-mm diameter temperature probe (model HYP-033-1-T-G-60-SMP-M; Omega Engineering) inserted 4.0 mm ventral to the surface of the skull. The probe was removed before the fluid percussion injury and replaced immediately after the injury. Rectal temperatures were measured with an electronic thermometer with an analog display (model 43 TE; YSI, Yellow Springs, OH) and a temperature probe (series 400; YSI). A brain temperature of 32 °C was achieved by immersing the body of the anesthetized rat in ice-cold water. The skin and fur of the animals were protected from direct contact with the water by placing each animal in a plastic bag (head exposed) before immersion. Animals were removed from the water bath when the brain temperature had dropped to within 2 °C of the target temperature. It took approximately 30 min to reach the target brain temperatures, which were maintained for 4 h under general anesthesia at room temperature by intermittent application of ice packs as needed. Gradual warming of the animals to normothermia levels (37 °C) was done over a 90-min period to avoid rapid warming that may have affected the secondary injury processes.

### Hematoxylin and eosin (HE) staining

Rats were subjected to deep anesthesia with 10% chloral hydrate. At 24 h after TBI, rats were perfused transcardially with 4% paraformaldehyde. The brains were removed, further fixed at 4 °C overnight, and then immersed in 30% sucrose/phosphate-buffered saline (PBS) at 4 °C overnight. Specimens were mounted in optimal cutting temperature compound (OCT). Serial sections were obtained by using a cryostat and were stained with toluidine blue for 30 min; two to three drops of glacial acetic acid were then added. Once the nucleus and granulation were clearly visible, the sections were mounted in Permount or Histoclad. Sections were cut in a microtome and adhered to glass slides with polylysine. Images of injured cortex and ipsilateral hippocampus were captured at × 100 by using a microscope (Nikon Labophot; Nikon USA, Melville, NY). There were six rats in each of the four groups.

### Immunohistochemical staining

The 4-mm-thick, formalin-fixed OCT-embedded sections were subjected to immunofluorescence analysis to determine the immunoreactivity of ionized calcium-binding adapter molecule 1 (Iba-1) and cleaved caspase 3. Endogenous peroxidase was blocked by treatment with 3% hydrogen peroxide for 5 min, followed by a brief rinse in distilled water and a 15-min wash in PBS. Sections were cooled at room temperature for 20 min and rinsed in PBS. Nonspecific protein binding was blocked by incubation in 5% horse serum for 40 min. Sections were incubated with primary antibodies (goat anti-rat Iba-1, diluted 1:100, Abcam; rabbit anti-rat cleaved caspase 3, diluted 1:100, CST) for 1 h at room temperature and then subjected to a 15-min wash in PBS. Sections were incubated with Alexa Fluor 488 donkey anti-goat secondary antibodies for Iba-1 and Alexa Fluor 555 donkey anti-rabbit secondary antibodies for cleaved caspase 3, protein light chain 3 (LC3), and Beclin-1 (1:1000 dilution, Invitrogen) for 1 h at room temperature. For negative controls, sections were incubated in the absence of a primary antibody. At least 10 randomly selected microscopic fields (× 630 magnification; Zeiss LSM880; Zeiss, Germany) were used for counting the Iba-1-positive and cleaved caspase 3-positive cells. There were six rats in each of the four groups.

### Immunofluorescence microscopy for cell localization

The primary antibodies for immunofluorescence were goat anti-rat Iba-1 (1:100 dilution, Abcam), rabbit anti-rat cleaved caspase 3 (1:100 dilution, CST), rabbit anti-rat LC3 (1:100 dilution, CST), and rabbit anti-rat Beclin-1 (1:100 dilution, Proteintech). The sections were incubated with the primary antibodies in PBS with 1% bovine serum albumin for 30–40 min at room temperature; this was followed by washing and application of the secondary antibodies. The secondary antibodies were Alexa Fluor 488 donkey anti-goat for Iba-1 (1:1000 dilution, Invitrogen) and Alexa Fluor 555 donkey anti-rabbit for cleaved caspase 3, LC3, and Beclin-1 (1:1000 dilution, Invitrogen). We performed double labeling for Iba-1/cleaved caspase 3, LC3, or Beclin-1 to detect expression of them in microglia. After a final wash, the sections were protected with cover slips with anti-fading mounting medium, sealed with clear nail polish, and stored at 4 °C for preservation. At least 10 randomly selected microscope fields were observed in each group, and the number of positive cells was statistically analyzed (× 630 magnifications; Zeiss LSM880; Zeiss, Germany). There were six rats in each of the four groups.

### Western blot analysis

At 24 h after TBI, the injured cortex and ipsilateral hippocampus were harvested. The frozen brain samples were mechanically lysed in 20 mM tris(hydroxymethyl)aminomethane (Tris; pH 7.6), containing 0.2% sodium dodecylsulfate (SDS), 1% Triton X-100, 1% deoxycholate, 1 mM phenylmethylsulphonyl fluoride, and 0.11 IU/mL aprotinin (all purchased from Sigma–Aldrich, Inc.). The lysates were centrifuged at 12,000*g* for 20 min at 4 °C. The protein concentration was estimated by the Bradford method. The samples (60 μg/lane) were separated by 12% SDS polyacrylamide gel electrophoresis and electro-transferred onto a polyvinylidene difluoride membrane (Bio-Rad Lab, Hercules, CA). The membrane was blocked with 5% skim milk for 2 h at room temperature and incubated with primary antibodies against TLR4 (1:1000 dilution, Proteintech), MyD88 (1:1000 dilution, Proteintech), cleaved caspase 3 (1:100 dilution, CST), and Iba-1 (1:1000 dilution, Abcam). β-Actin (1:10,000 dilution, Sigma–Aldrich) was used as the loading control. After the membrane had been washed six times in a mixture of Tris-buffered saline and Tween-20 (TBST) for 10 min each time, it was incubated with the appropriate horseradish peroxidase-conjugated secondary antibody (1:10,000 dilution in TBST) for 2 h. The blotted protein bands were visualized by enhanced chemiluminescence Western blot detection reagents (Amersham, Arlington Heights, IL) and exposed to X-ray film. The developed films were digitized using an Epson Perfection 2480 scanner (Seiko Corp, Nagano, Japan). The results were quantified by Quantity One Software (Bio-Rad). The band density values were calculated as a ratio of TLR4, MyD88, Iba-1, and cleaved caspase 3/β-actin. There were six rats in each of the four groups.

### Quantitative real-time polymerase chain reaction (qRT-PCR)

Total RNA was isolated from the injured cortex and the ipsilateral hippocampus by using Trizol (Invitrogen) according to the manufacturer’s instructions. cDNA was synthesized by using a reverse transcription kit (TAKARA). PCR was performed by using SYBR Advantage Premix (TAKARA). The primers for TLR4 were (forward) 5′-TGT TCC TTT CCT GCC TGA GAC-3′ and (reverse) 5′-GGT TCT TGG TTG AAT AAG GGA TGT C-3′. The primers for MyD88 were (forward) 5′-GGT TCT GGA CCC GTC TTG C-3′ and (reverse) 5′-AGA ATC AGG CTC CAA GTC AGC-3′. Relative mRNA expression was calculated with the 2^−ΔΔCt^ method; SNG values were taken as 100%. β-Actin was used as the control. All experiments were done in triplicate. There were six rats in each of the four groups.

### Enzyme-linked immunosorbent assay (ELISA) analysis of TNF-α and interleukin-1β (IL-1β)

At 24 h after TBI, rats were subjected to deep anesthesia by 10% chloral hydrate. The brains were quickly removed by dissection and kept over ice in physiologic salt solution. The injured cortex and ipsilateral hippocampus specimens were separated, cut into small pieces, dispersed by aspiration into a pipette, and suspended in 1 mL of physiologic salt solution in a test tube. Samples were kept over wet ice for 20 min before use. The homogenates were centrifuged at 7500 rpm for 20 min. The supernatants were used for measuring TNF-α and IL-1β concentrations with commercial ELISA kits (Shanghai Enzyme-linked Biotechnology Co., Ltd.) by following the manufacturer’s instructions. There were six rats in each of the four groups.

### Statistical analysis

All data are presented as the mean ± the standard deviation (SD). SPSS for Windows version 23.0 (SPSS, Inc., Chicago, IL) was used for statistical analysis of the data. All data were subjected to one-way analysis of variance. Post hoc comparisons were made with Fisher’s least significant difference test. Statistical significance was inferred at *P* < 0.05.

## Results

### Histologic examination of the injured cortex and the ipsilateral hippocampus

**Fig. 1:**
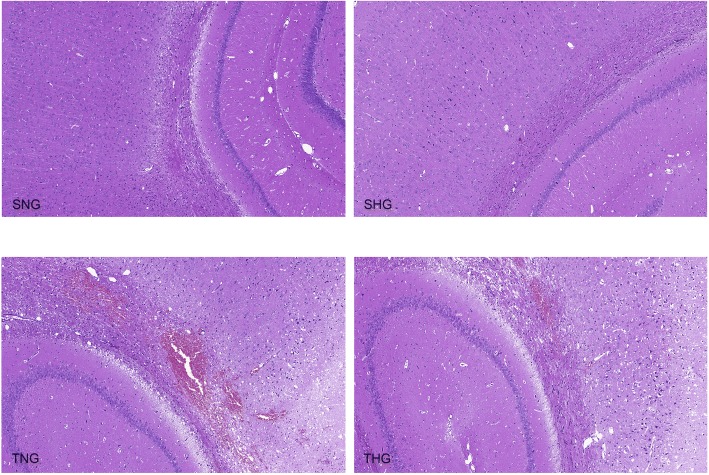
HE staining of the injured cortex and ipsilateral hippocampus. HE staining of the injured cortex and ipsilateral hippocampus from SNG, SHG, TNG, and THG rats 24 h after TBI (magnification, × 100). The gray/white matter interface shows visible contusion and hemorrhaging in the TNG and THG rats

The brains of SNG and SHG rats showed the normal neuronal structure. The gray/white matter interface showed visible contusions and hemorrhaging in TNG and THG rats (Fig. 1).

### TBI caused cell apoptosis, microglial activation, and an inflammatory response, which moderate hypothermia inhibited

**Fig. 2:**
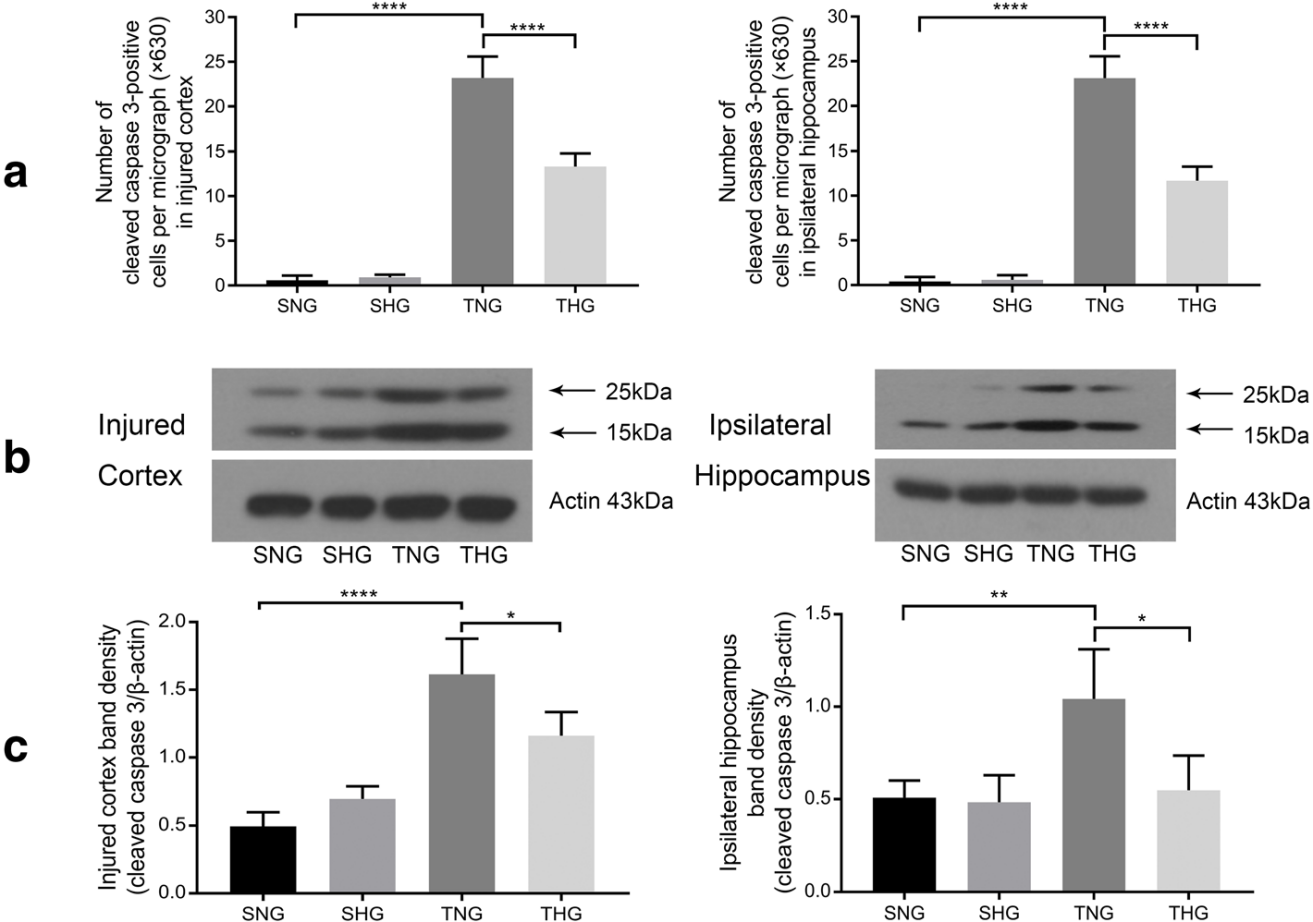
Immunofluorescence analysis and Western blotting of cleaved caspase 3 expression in the injured cortex and ipsilateral hippocampus. **a** Number of cleaved caspase 3-positive cells in the injured cortex and ipsilateral hippocampus. Data in the bar graphs represent mean ± SD. ****P < 0.001. At least 10 randomly selected microscopic fields were used for counting (magnification, × 630). **b**, **c** Western blotting of cleaved caspase 3 from the injured cortex and ipsilateral hippocampus. Data in the bar graphs represent mean ± SD. β-Actin was used as the load control. *P < 0.05; **P < 0.01; ****P < 0.001

Immunohistochemical staining and Western blotting of cleaved caspase 3 were used to evaluate cell apoptosis in injured cortex and ipsilateral hippocampus samples. Few cleaved caspase 3-positive cells were found in both areas in SNG and SHG rats. At 24 h after TBI, the expression level of cleaved caspase 3-positive cells significantly increased relative to those in SNG rats (TNG: immunohistochemical staining: injured cortex, 23.2 ± 2.39, *P* < 0.001; ipsilateral hippocampus, 23.1 ± 2.47, *P* < 0.001, and Western blotting: injured cortex, 1.61 ± 0.26, *P* < 0.001; ipsilateral hippocampus, 1.04 ± 0.27, *P* < 0.01). However, moderate hypothermia inhibited cell apoptosis in comparison with that in TNG rats (THG: immunohistochemical staining: injured cortex, 13.3 ± 1.49, *P* < 0.001; ipsilateral hippocampus, 11.70 ± 1.57, *P* < 0.001, and Western blotting: injured cortex, 1.16 ± 0.18, *P* < 0.05; ipsilateral hippocampus, 0.55 ± 0.19, *P* < 0.05). There were no significant differences between the SNG and SHG rats (Fig. 2 and Additional file 1: Figure S1).

**Fig. 3:**
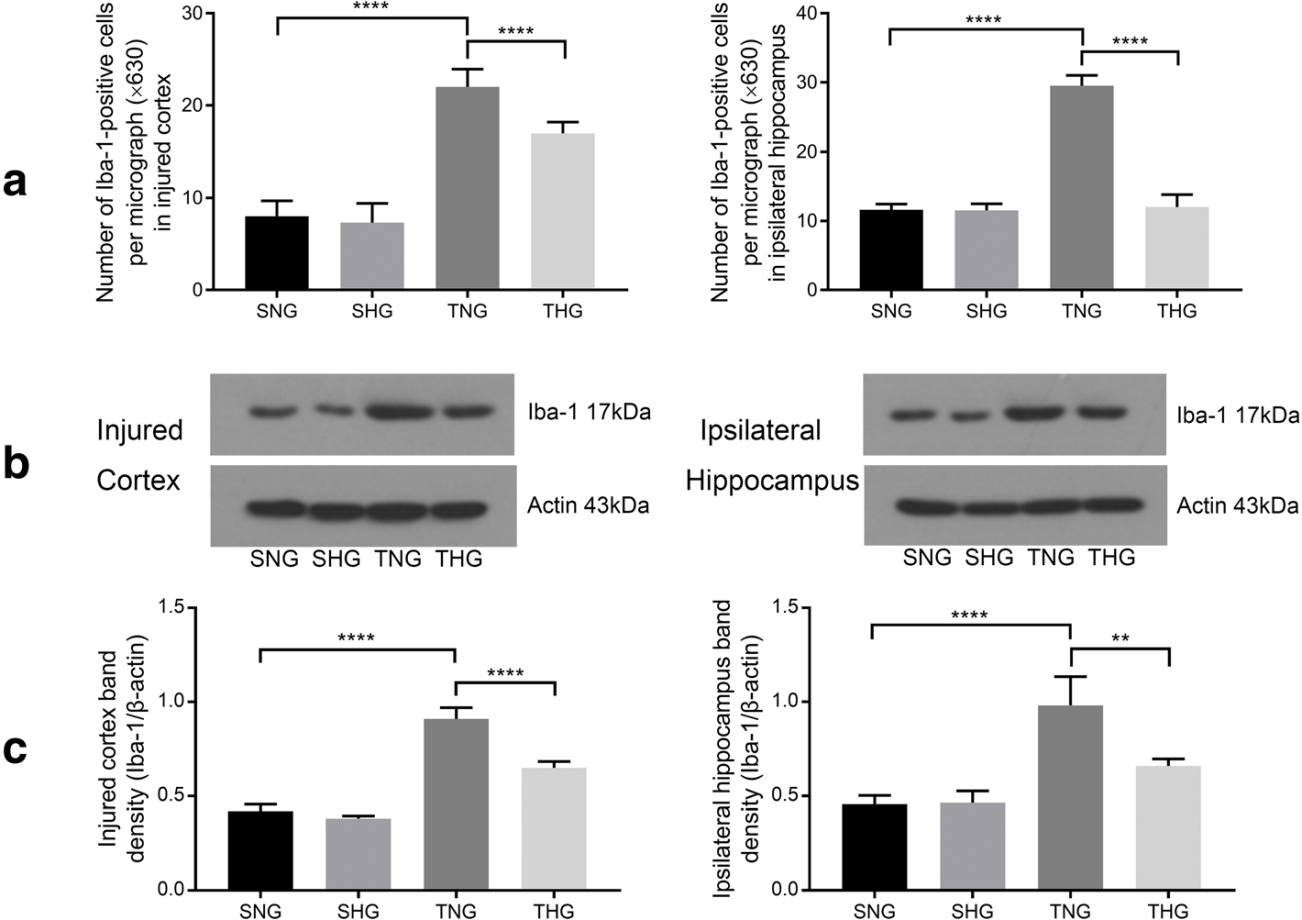
Immunofluorescence analysis and Western blotting of Iba-1 expression in the injured cortex and ipsilateral hippocampus. **a** Number of Iba-1-positive cells in the injured cortex and ipsilateral hippocampus. Data in the bar graphs represent mean ± SD. ****P < 0.001. At least 10 randomly selected microscopic fields were used for counting (magnification, × 630). **b**, **c** Western blotting of Iba-1 from the injured cortex and ipsilateral hippocampus. Data in the bar graphs represent mean ± SD. β-Actin was used as the load control. **P < 0.01; ****P < 0.001

Changes in microglial activation were evaluated from differences in Iba-1 expression, as determined by immunohistochemical staining and Western blotting of Iba-1. The expression level of Iba-1 significantly increased 24 h after TBI (TNG: immunohistochemical staining: injured cortex, 22 ± 1.94, *P* < 0.001; ipsilateral hippocampus, 29.5 ± 1.51, *P* < 0.001, and Western blotting: injured cortex, 0.91 ± 0.06, *P* < 0.001; ipsilateral hippocampus, 0.98 ± 0.15, *P* < 0.001), whereas moderate hypothermia inhibited microglial activation in comparison with that in TNG rats (THG: immunohistochemical staining: injured cortex, 17 ± 1.25, *P* < 0.001; ipsilateral hippocampus, 12 ± 1.83, *P* < 0.001, and Western blotting: injured cortex, 0.65 ± 0.03, *P* < 0.001; ipsilateral hippocampus, 0.66 ± 0.04, *P* < 0.01). There were no significant differences between SNG and SHG rats (Fig. 3 and Additional file 2: Figure S2).

**Fig. 4:**
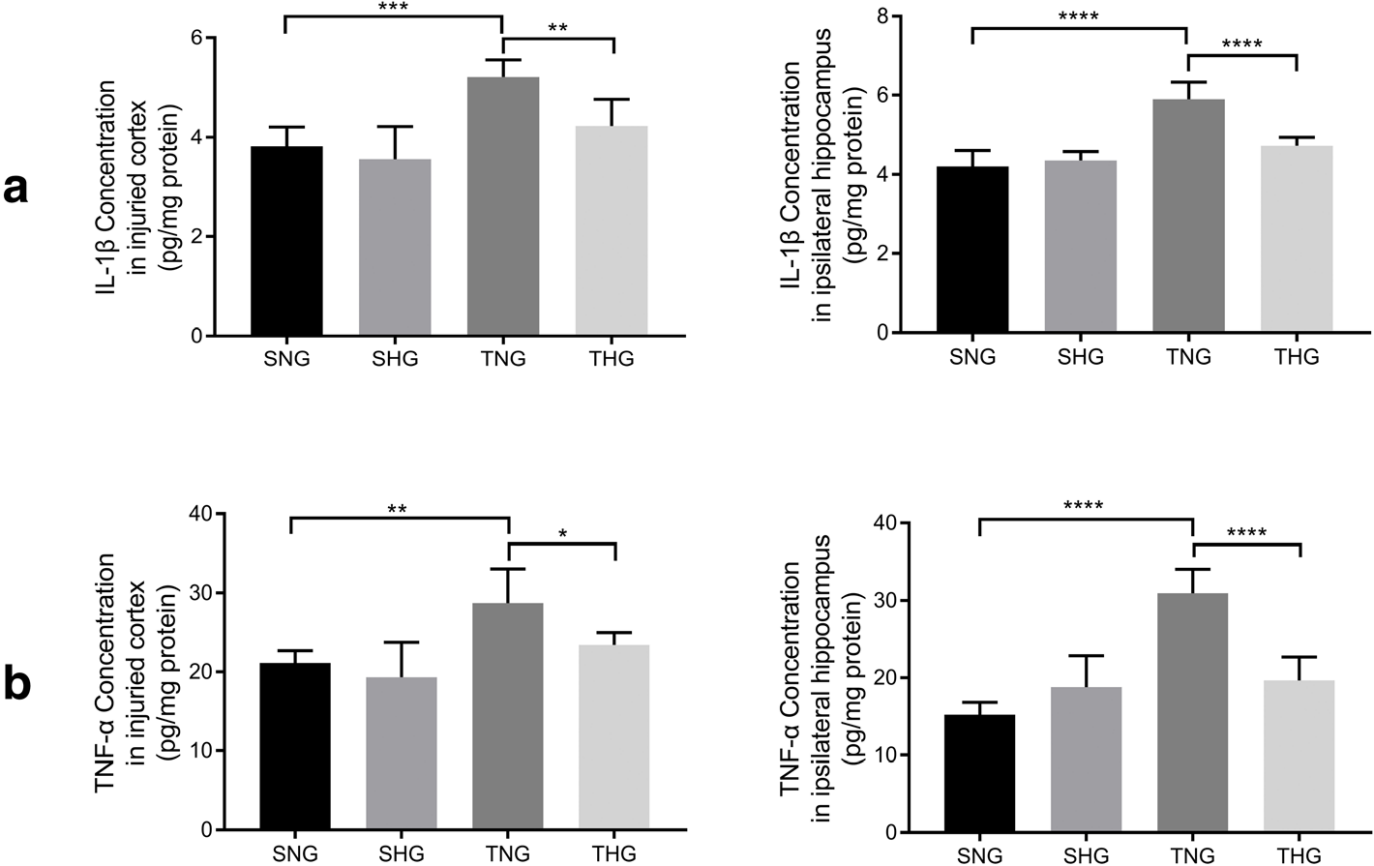
ELISA of IL-1β and TNF-α from the injured cortex and ipsilateral hippocampus. The levels of IL-1β (**a**) and TNF-α (**b**) from the injured cortex and ipsilateral hippocampus with or without moderate hypothermia are shown. Data in the bar graphs represent mean ± SD. *P < 0.05; **P < 0.01; ***P < 0.005; ****P < 0.001. There were six rats in each of the four groups

TNF-α and IL-1β are involved in the inflammatory response after TBI. To determine the differences in the inflammatory responses, we separately tested the expression of TNF-α and IL-1β in the injured cortex and ipsilateral hippocampus. The results showed that the expression levels of TNF-α and IL-1β in TNG rats were significantly increased relative to those in SNG rats (TNG: injured cortex: TNF-α, 28.73 ± 4.33, *P* < 0.01; IL-1β, 5.21 ± 0.34, *P* < 0.005, and ipsilateral hippocampus: TNF-α, 30.95 ± 3.09, *P* < 0.001; IL-1β, 5.90 ± 0.43, *P* < 0.001). Moderate hypothermia attenuated the expression of TNF-α and IL-1β in comparison with that in TNG rats (THG: injured cortex: TNF-α, 23.41 ± 1.58, *P* < 0.05; IL-1β, 4.23 ± 0.53, *P* < 0.01, and ipsilateral hippocampus: TNF-α, 19.64 ± 3.05, *P* < 0.001; IL-1β, 4.73 ± 0.21, *P* < 0.001). There were no significant differences between SNG and SHG rats (Fig. 4).

### TBI caused microglial autophagy, which moderate hypothermia attenuated

**Fig. 5:**
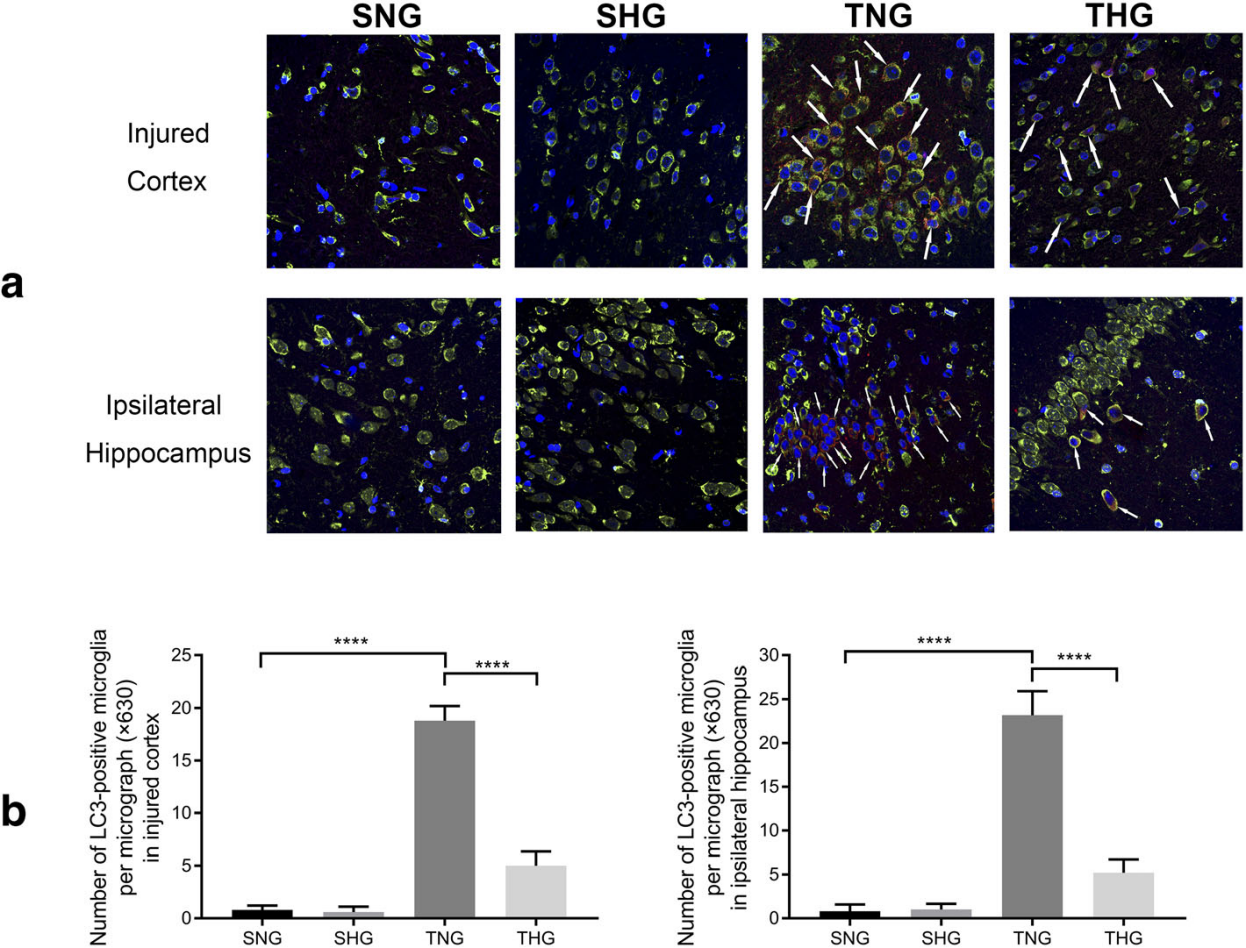
Immunofluorescence analysis of LC3 (red) and Iba-1 (green) from the injured cortex and ipsilateral hippocampus. **a** Immunohistochemical staining of LC3 and Iba-1 from the injured cortex and ipsilateral hippocampus. Arrows indicate co-localization of LC3 and Iba-1 (magnification, × 630). **b** Number of LC3-positive microglia in the injured cortex and ipsilateral hippocampus. At least 10 randomly selected microscopic fields were used for counting. Data in the bar graphs represent mean ± SD. ****P < 0.001

**Fig. 6:**
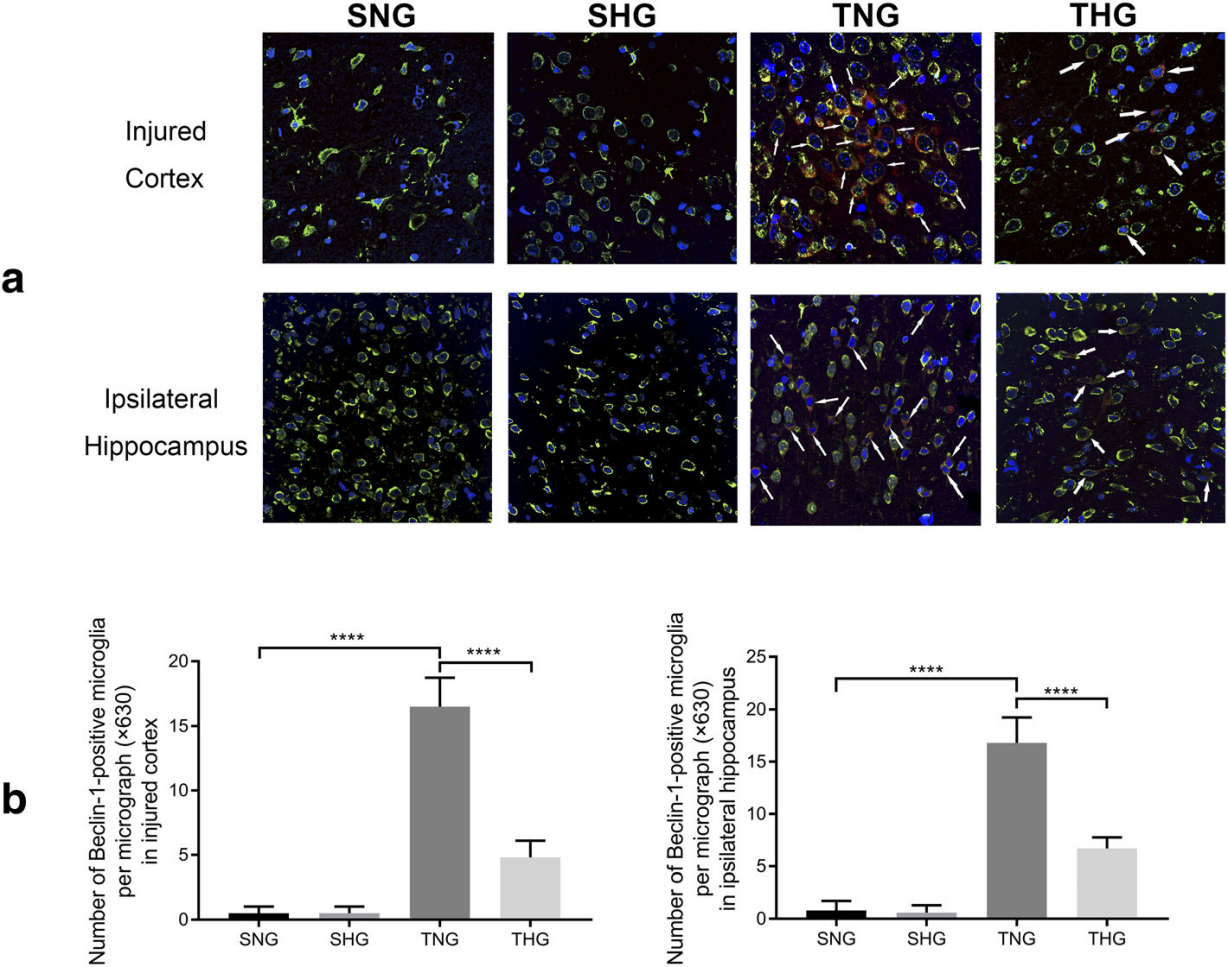
Immunofluorescence analysis of Beclin-1 (red) and Iba-1 (green) from the injured cortex and ipsilateral hippocampus. **a** Immunohistochemical staining of Beclin-1 and Iba-1 from the injured cortex and ipsilateral hippocampus. Arrows indicate co-localization of Beclin-1 and Iba-1 (magnification, × 630). **b** Number of Beclin-1-positive microglia in the injured cortex and ipsilateral hippocampus. At least 10 randomly selected microscopic fields were used for counting. Data in the bar graphs represent mean ± SD. ****P < 0.001

A few LC3- and Beclin-1-positive microglial cells were observed in the sham groups and indicated the constitutive activity of autophagy in the normal rat brain. At 24 h after TBI, the LC3- and Beclin-1-positive microglial cells in the injured cortex and ipsilateral hippocampus were significantly increased in number compared with those in SNG rats (TNG: LC3-positive microglia: injured cortex, 18.8 ± 1.4, *P* < 0.001; ipsilateral hippocampus, 23.2 ± 2.74, *P* < 0.001, and Beclin-1-positive microglia: injured cortex, 16.5 ± 2.22, *P* < 0.001; ipsilateral hippocampus, 16.8 ± 2.44, *P* < 0.001). However, moderate hypothermia significantly attenuated microglial autophagy relative to that in TNG rats (THG: LC3-positive microglia: injured cortex, 5 ± 1.33, *P* < 0.001; ipsilateral hippocampus, 5.2 ± 1.55, *P* < 0.001, and Beclin-1-positive microglia: injured cortex, 4.8 ± 1.32, *P* < 0.001; ipsilateral hippocampus, 6.7 ± 1.06, *P* < 0.001). There were no significant differences between SNG and SHG rats (Figs. 5 and 6).

### TBI caused microglial apoptosis, which moderate hypothermia promotion

**Fig. 7:**
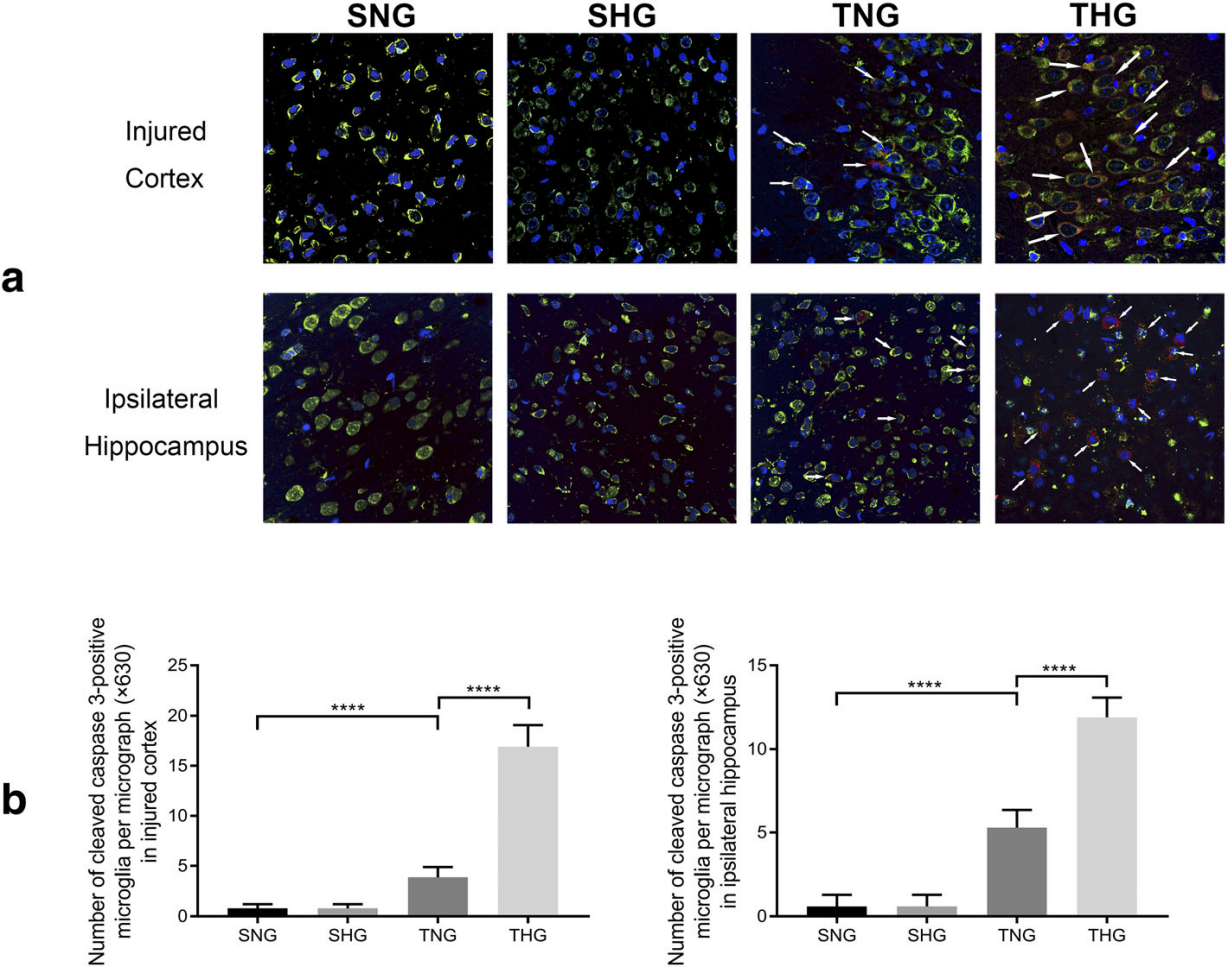
Immunofluorescence analysis of cleaved caspase 3 (red) and Iba-1 (green) from the injured cortex and ipsilateral hippocampus. **a** Immunohistochemical staining of cleaved caspase 3 and Iba-1 from the injured cortex and ipsilateral hippocampus. Arrows indicate co-localization of cleaved caspase 3 and Iba-1 (magnification, × 630). **b** Number of cleaved caspase 3-positive microglia in the injured cortex and ipsilateral hippocampus. At least 10 randomly selected microscopic fields were used for counting. Data in the bar graphs represent mean ± SD. ****P < 0.001

Induction of microglial apoptosis was detected with immunohistochemical staining of Iba-1 and cleaved caspase 3. The number of cleaved caspase 3-positive microglial cells significantly increased 24 h after TBI in the injured cortex and ipsilateral hippocampus (TNG: injured cortex, 3.9 ± 0.99, *P* < 0.001; ipsilateral hippocampus, 5.3 ± 1.06, *P* < 0.001), whereas moderate hypothermia promoted microglial apoptosis, relative to that in TNG rats (THG: injured cortex, 16.9 ± 2.18, *P* < 0.001; ipsilateral hippocampus, 11.9 ± 1.20, *P* < 0.001). There were no significant differences between SNG and SHG rats (Fig. 7).

### TBI activated the MyD88-dependent TLR4 pathway, which was inhibited by moderate hypothermia

**Fig. 8:**
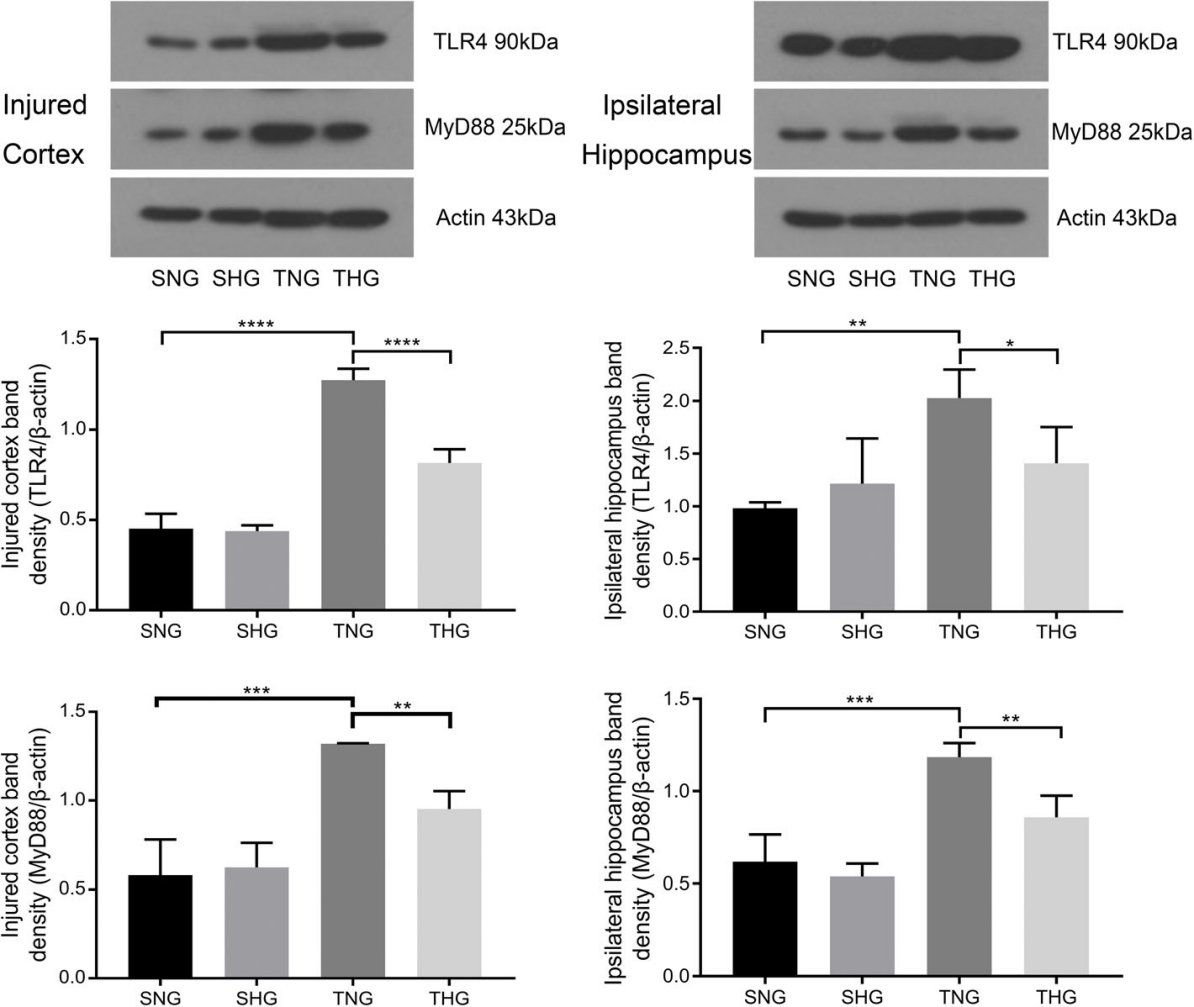
Western blotting of TLR4 and MyD88 from the injured cortex and ipsilateral hippocampus. Data in the bar graphs represent mean ± SD. β-Actin was used as the load control. *P < 0.05; **P < 0.01; ***P < 0.005; ****P < 0.001

Expression of TLR4 and MyD88 in microglial cells was measured with Western blotting and qRT-PCR. The protein levels of TLR4 and MyD88 significantly increased in the injured cortex and ipsilateral hippocampus 24 h after TBI (TNG: TLR4: injured cortex, 1.27 ± 0.06, *P* < 0.001; ipsilateral hippocampus, 2.03 ± 0.27, *P* < 0.01, and MyD88: injured cortex, 1.32 ± 0.002, *P* < 0.005; ipsilateral hippocampus, 1.18 ± 0.08, *P* < 0.005). Moderate hypothermia decreased the protein levels of TLR4 and MyD88 relative to those in TNG rats (THG: TLR4: injured cortex, 0.82 ± 0.08, *P* < 0.001; ipsilateral hippocampus, 1.41 ± 0.34, *P* < 0.05, and MyD88: injured cortex, 0.95 ± 0.10, *P* < 0.01; ipsilateral hippocampus, 0.86 ± 0.12, *P* < 0.01). There were no significant differences between SNG and SHG rats (Fig. 8).

**Fig. 9:**
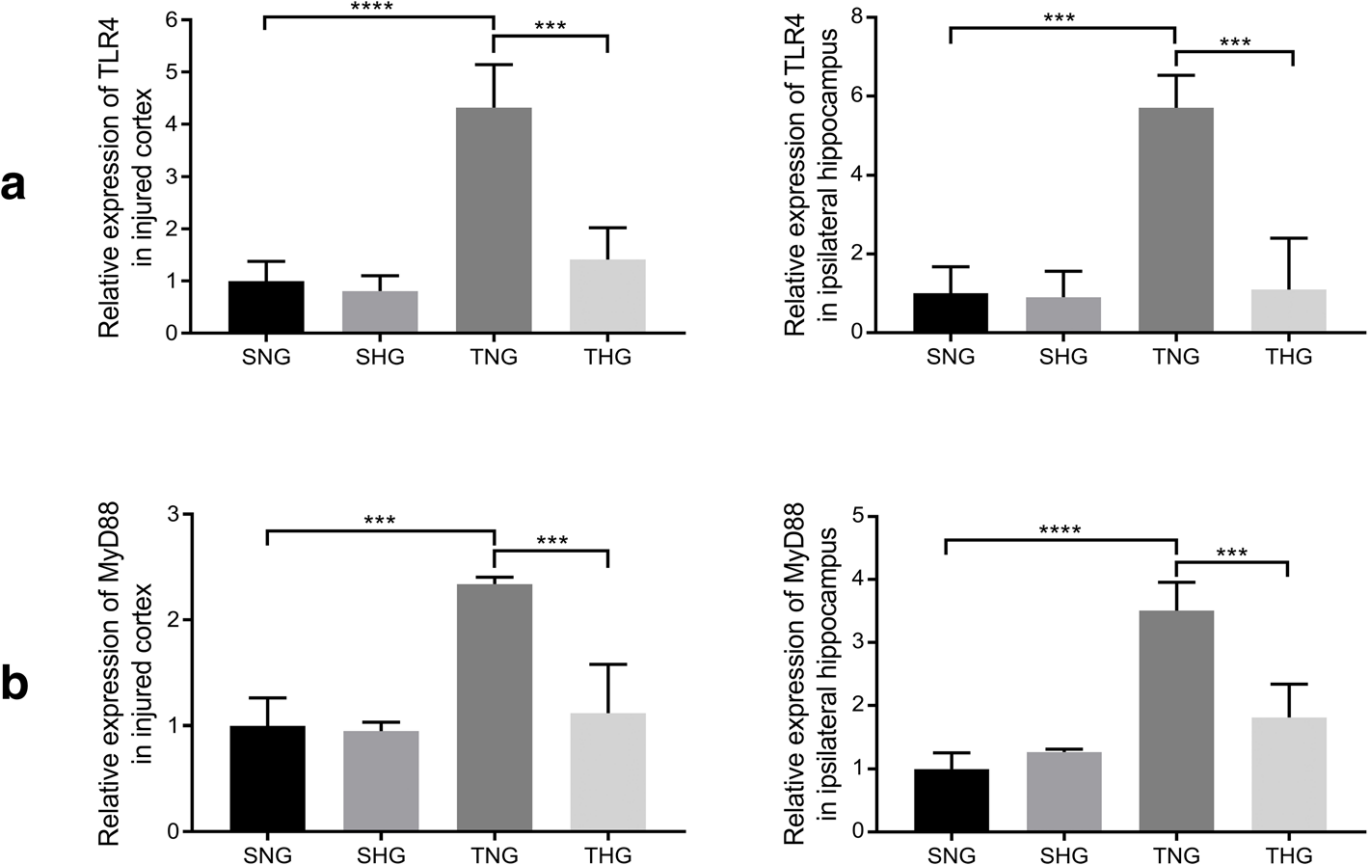
qRT-PCR of TLR4 and MyD88 from the injured cortex and ipsilateral hippocampus. Changes in the expression of TLR4 (**a**) and MyD88 (**b**) are shown as *n*-fold. Data in the bar graphs represent mean ± SD. ***P < 0.005; ****P < 0.001. There were six rats in each of the four groups

Meanwhile, the mRNA levels of TLR4 and MyD88 in the injured cortex and ipsilateral hippocampus also significantly increased 24 h after TBI (TNG: TLR4: injured cortex, 4.3-fold, *P* < 0.001; ipsilateral hippocampus, 5.7-fold, *P* < 0.005, and MyD88: injured cortex, 2.3-fold, *P* < 0.005; ipsilateral hippocampus, 3.5-fold, *P* < 0.001). Moderate hypothermia also decreased the mRNA levels of TLR4 and MyD88 compared with those in TNG rats (TLR4: injured cortex, 1.4-fold, *P* < 0.005; ipsilateral hippocampus, 1.1-fold, *P* < 0.005, and MyD88: injured cortex, 1.1-fold, *P* < 0.005; ipsilateral hippocampus, 1.8-fold, *P* < 0.005). There were no significant differences between SNG and SHG rats (Fig. 9).

## Discussion

Our results suggest that microglial activation post-TBI could be suppressed by moderate hypothermia and that the negative regulation of autophagy and apoptosis may play a role in this process. In addition, hypothermia may also act by inhibiting the MyD88-dependent TLR4 pathway. Thus, moderate hypothermia exerts anti-inflammatory and neuroprotective effects.

In this study, we found that moderate TBI induced by fluid percussion caused cortical and ipsilateral hippocampal cell death, microglial activation, and microglial autophagy 24 h after TBI. We also found that moderate hypothermia could reduce the amount of hippocampal and cortical cell death. Furthermore, we found that microglial autophagy was suppressed, and microglial apoptosis was increased after moderate hypothermia 24 h post-TBI. In addition, the attenuation of microglial activation was observed through the downregulated expression of Iba-1, which is an important biomarker for microglial activation. These results suggest that moderate hypothermia may reduce the number of activated microglia by inhibiting autophagy and promoting apoptosis.

Apoptotic cell death is one of the most common pathologic changes after TBI [15–18]. The activation of cleaved caspase 3, which is an executioner caspase, presents an irreversible point in the complex cascade of apoptosis induction. Activated caspase-3 has been detected in neurons, astrocytes, and oligodendrocytes post-TBI in previous studies [19–23]. In this study, the number of microglia labeled by cleaved caspase-3 significantly increased after moderate hypothermia post-TBI, which indicates that hypothermia accelerated apoptosis of the microglia.

Autophagy is a highly regulated process involving the bulk degradation of cytoplasmic macromolecules and organelles in mammalian cells through the lysosomal system. Beclin-1, an autophagic biomarker, is a novel Bcl-2-homology-3 domain-only protein that participates in autophagy regulation with several co-actors [24]. LC3, a mammalian orthologue of yeast ATG8, is synthesized as pro-LC3, which is cleaved by ATG4 protease and converted into LC3-I. Once autophagy is activated, LC3-I is conjugated to phosphatidylethanolamine (lipidated) to form LC3-II. The amounts of LC3-II and p62 degraded by autophagy provide an estimate of the autophagy activity [25]. Diskin et al. first demonstrated that the Beclin-1 level increased near the site of an injury by using the closed-head injury model in mice in 2005 [26]. Viscomi et al. found that autophagy could serve as a protective mechanism for maintaining cellular homeostasis after TBI, which could be enhanced by rapamycin through inactivation of the mammalian target of rapamycin [27]. However, the role of autophagy is still controversial. In previous studies, we have demonstrated that moderate hypothermia post-TBI could activate the neuronal and glial autophagy pathway, which could negatively modulate apoptosis and reduce cell death [8, 14]. On the other hand, this negative regulatory effect of autophagy on apoptosis may play a different role in microglia and may be associated with different cell types. In this study, the use of LC3 and Beclin-1 as autophagic biomarkers showed that microglial autophagy could be inhibited by moderate hypothermia.

From the above discussion, it can be stated that moderate hypothermia reduces the number of activated microglia. Moderate hypothermia inhibits autophagy and promotes apoptosis, which may be the possible mechanism. However, how moderate hypothermia specifically affects the autophagy and apoptosis of microglia post-TBI is not very clear. This issue needs further research.

In addition to reducing the number of activated microglia, the moderate hypothermia may also directly affect the microglial pro-inflammatory function. In the current study, we found that a moderate TBI induced by fluid percussion led to high expression of the inflammatory cytokines TNF-α and IL-1β. We also found that moderate hypothermia post-TBI could inhibit the expression of TLR4 and MyD88 and reduce the level of the inflammatory cytokines TNF-α and IL-1β after 24 h. This suggested that activated microglia might initiate neuroinflammatory responses post-TBI through the MyD88-dependent TLR4 pathway. Moderate hypothermia may reduce the release of inflammatory cytokines from activated microglia by inhibiting the MyD88-dependent TLR4 pathway.

Toll-like receptors play a role in microglial activation, and toll-like receptor-associated pathway mediates the release of pro-inflammatory cytokines [28]. TLR4 is the most abundant toll-like receptor expressed in microglia [10, 11]. TLR4 could activate IRAK and TRAF6 via the MyD88-dependent pathway. TRAF6 induces the activation of transforming growth factor-β-activated kinase 1, which leads to the activation of the mitogen-activated protein kinase and IκB kinase (IKK) cascades [29, 30]. When activated by these signals, IKK phosphorylates two serine residues located in an IκB regulatory domain, and the IκB proteins are ubiquitinated and degraded by proteasomes. After that, the NF-κB complex is freed to enter the nucleus, where it can further induce the expression of pro-inflammatory cytokines IL-6, TNF-α, IL-12, and so on [12, 13, 31, 32]. This current study demonstrated that moderate hypothermia might decrease the level of inflammatory cytokines by inhibiting the expression of relevant proteins in the MyD88-dependent TLR4 pathway.

Iba-1, also known as allograft inflammatory factor 1, is a 17-kDa EF-hand protein that is specifically expressed in macrophages/microglia and is upregulated during the activation of these cells. Ionized calcium-binding adapter molecule 1 (Iba1) expression is upregulated in microglia following nerve injury [33]. There is a constitutive expression of Iba-1 in microglial cells, but Iba-1 has also been regarded in several articles as an important biomarker to detect the activation of microglia [34–36]. After referring to these papers, we chose to detect the expression level of Iba-1 for microglial activation by immunohistochemical staining and Western blotting. However, Iba-1 is not an ideal biomarker to distinguish microglial cells from macrophages. Other biomarkers, such as CD11b and CD68, are not of high specificity to microglia. The key to studying microglial activation after TBI is to figure out the immune subtypes of microglia. Therefore, we are planning to define the subtypes of macrophages/microglia post-TBI with a series of biomarkers in animal models in the future.

In previous studies, we estimated the role of autophagy and apoptosis in neuron and glial cells post-TBI, as well as the modulation of moderate hypothermia upon them. The term “glial cells” includes both macroglia and microglia. Macroglia, including oligodendrocytes, astrocytes, and ependymal cells, are derived from ectodermal tissues. However, microglia are derived from the earliest wave of mononuclear cells that originate in the yolk sac blood islands early in development. Microglia are resident immune cells in the central nervous system. In our opinion, microglia are distinct from macroglia, although they can be classified as glial cells. The current study is based on this opinion and extends the research that has taken place in the past. The effects of moderate hypothermia post-TBI are quite different between microglia and macroglia, which is our core concern. This study initially explored the changes in microglial autophagy and apoptosis after TBI and the regulation of hypothermia upon these processes. This study only draws preliminary conclusions.

The specific mechanism of moderate hypothermia on the TLR4 pathway of microglia post-TBI remains unclear. Logically, it would be necessary to inhibit or block the TLR4/MyD88 pathway to study the effects of hypothermia on microglial activation. But this pathway is quite important, and we are concerned that inhibition or knockout of this pathway may affect the survival rate of the experimental animals after TBI. Therefore, we are planning to establish an in vitro model of a cell stretch injury. We can then perform more efficient gene editing on microglia and study the specific mechanism of moderate hypothermia on the TLR4 pathway.

The negative correlation between autophagy and apoptosis has been widely observed in our past studies and in this study. The autophagy inhibitor 3-methyladenine (3-MA) was used after moderate hypothermia post-TBI in a previous report [9], and an increase of apoptosis was observed therewith. Therefore, we preliminarily speculated that there is a negative modulation between autophagy and apoptosis. We intend to establish in vitro or in vivo models of TBI in the future and perform moderate hypothermia on microglia with and without addition of 3-MA, to further investigate the effect of hypothermia on the pathological process of autophagy and apoptosis.

## Conclusion

In the present study, microglial activation could be induced by TBI and moderate hypothermia could reduce the number of activated microglia by inhibiting autophagy and promoting apoptosis, probably through negative modulation between autophagy and apoptosis. In addition, moderate hypothermia may attenuate the pro-inflammatory function of microglia by inhibiting the MyD88-dependent TLR4 signaling pathway. This study preliminarily elucidates the possible molecular mechanism for participation of microglia in the neuroprotective effect of post-TBI moderate hypothermia and suggests new ideas for the investigation of efficacious neuroprotective methods. Further research is needed to deeply investigate the effect of moderate hypothermia on microglia after traumatic brain injury and the underlying molecular mechanism behind it.

## Additional files

Additional file 1: **Figure S1**. Immunofluorescence photos of cleaved caspase 3 expression in the injured cortex and ipsilateral hippocampus. Arrows indicate cleaved caspase 3-positive cells (magnification, × 630). (JPG 5698 kb)

Additional file 2: **Figure S2.** Immunofluorescence photos of Iba-1 expression in the injured cortex and ipsilateral hippocampus. Arrows indicate Iba-1-positive cells (magnification, × 630). (JPG 6548 kb)

## Abbreviations

3-MA: 3-Methyladenine
ELISA: Enzyme-linked immunosorbent assay
HE: Hematoxylin and eosin
i.p.: Intraperitoneal injection
Iba1: Ionized calcium-binding adapter molecule 1
IKK: IκB kinase
IL-12: Interleukin 12
IL-1β: Interleukin 1β
IL-6: Interleukin 6
LC3: Protein light chain 3
MyD88: Myeloid differentiation primary response 88
NF-κB: Nuclear factor kappa light chain enhancer of activated B cells
OCT: Optimal cutting temperature compound
PBS: Phosphate-buffered saline
qRT-PCR: Quantitative real-time polymerase chain reaction
SD: Standard deviation
SDS: Sodium dodecylsulfate
SHG: Sham injury with hypothermia group
SNG: Sham injury with normothermia group
TBI: Traumatic brain injury
TBST: Tris-buffered saline and Tween-20
THG: TBI with hypothermia group
TLR4: Toll-like receptor 4
TNF-α: Tumor necrosis factor-α
TNG: TBI with normothermia group
TRAF6: Adaptor molecules TNF receptor-associated factor 6
Tris: Tris(hydroxymethyl)aminomethane
