# Supplementary figures and images for: Moderate hypothermia inhibits microglial activation after traumatic brain injury by modulating autophagy/apoptosis and the MyD88-dependent TLR4 signaling pathway
Source: J Neuroinflammation. 2018 Sep 20;15:273. doi: 10.1186/s12974-018-1315-1 (PMC6146525; doi:10.1186/s12974-018-1315-1)

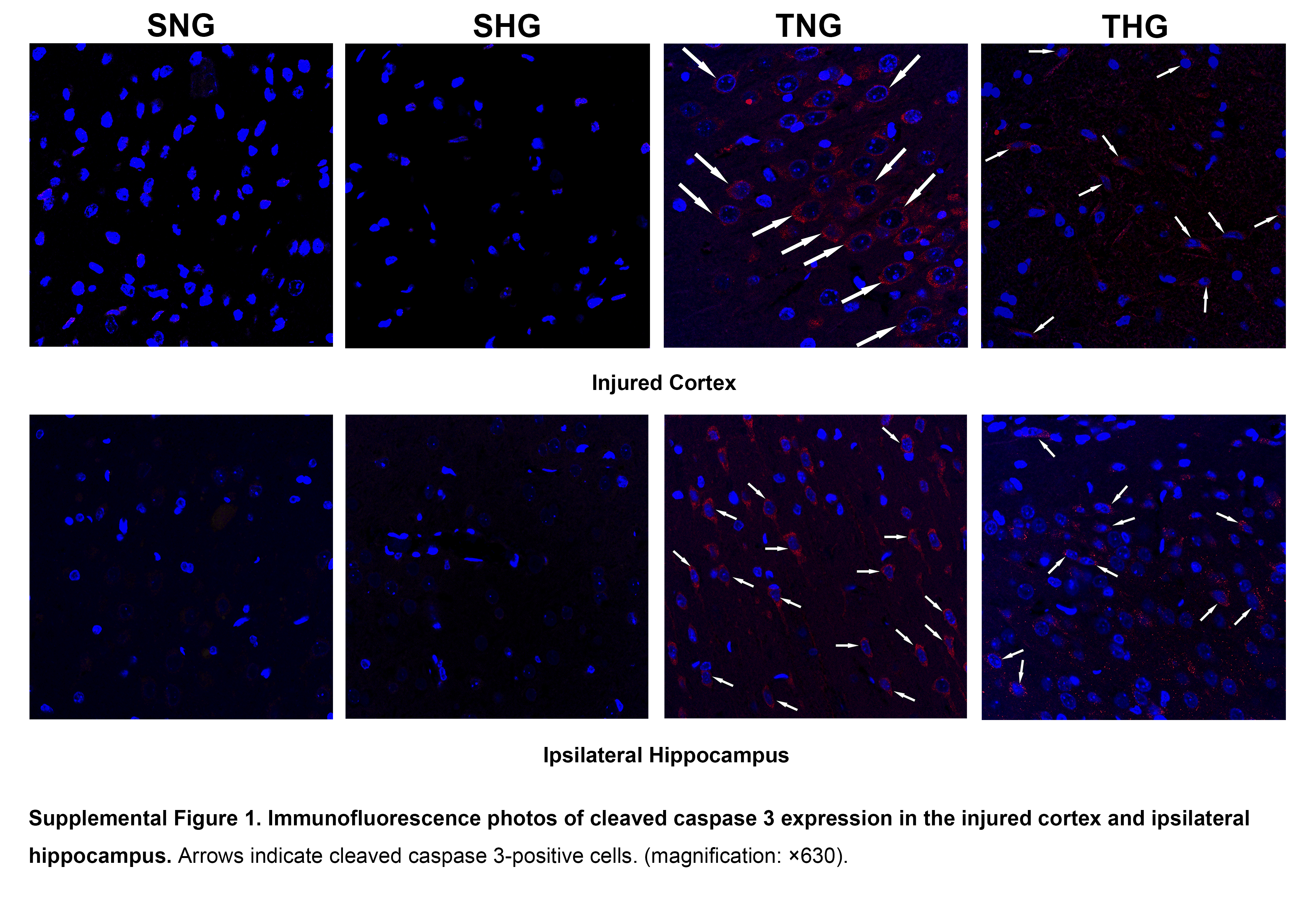

Supplement: Supplementary file 1 — Figure S1. Immunofluorescence photos of cleaved caspase 3 expression in the injured cortex and ipsilateral hippocampus. Arrows indicate cleaved caspase 3-positive cells (magnification, × 630). (JPG 5698 kb) [file 12974_2018_1315_MOESM1_ESM.jpg]

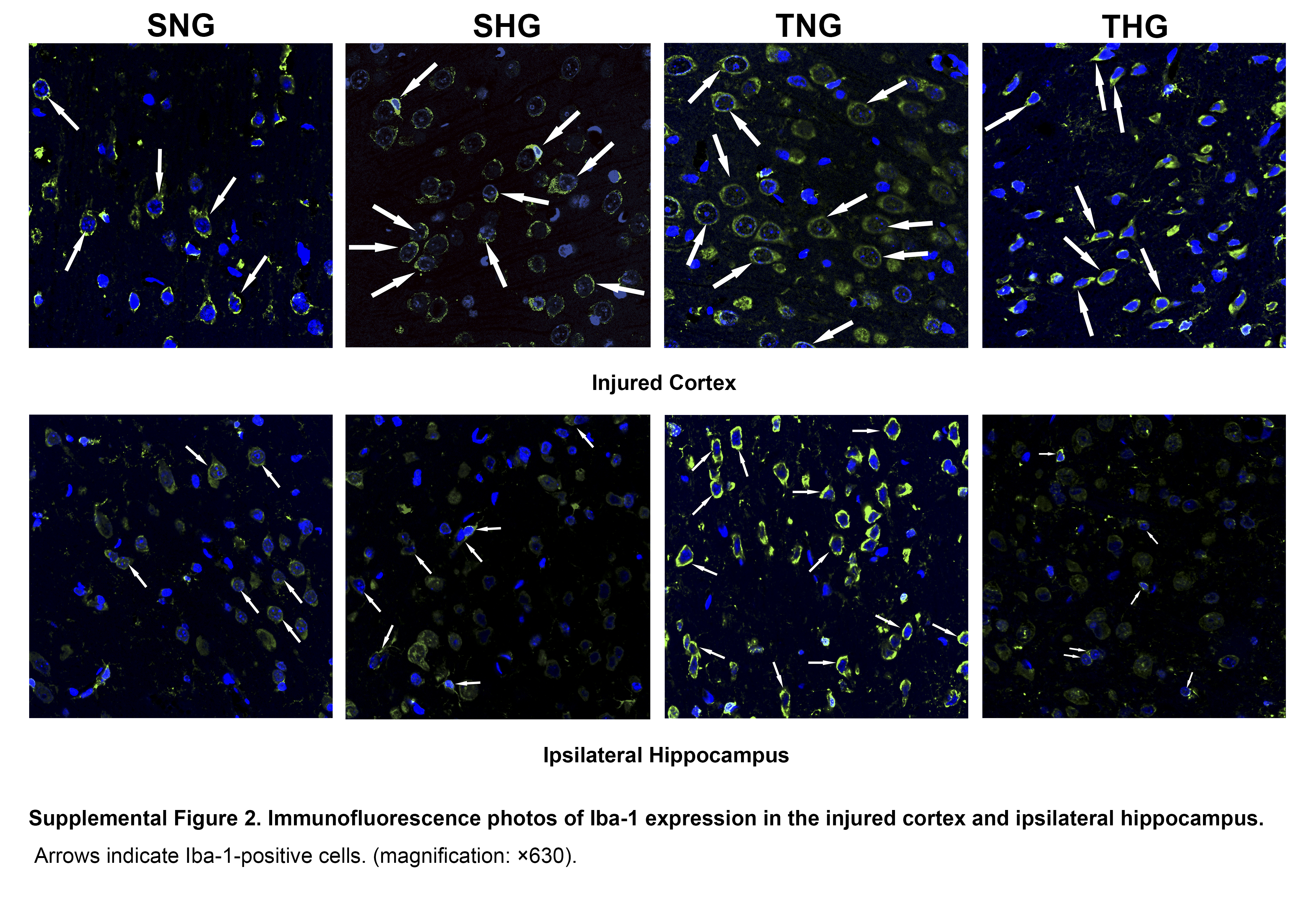

Supplement: Supplementary file 2 — Figure S2. Immunofluorescence photos of Iba-1 expression in the injured cortex and ipsilateral hippocampus. Arrows indicate Iba-1-positive cells (magnification, × 630). (JPG 6548 kb) [file 12974_2018_1315_MOESM2_ESM.jpg]
